# Genotypic Profiling of Virulence and Antibiotic Resistance Genes of *Escherichia coli* Isolates Derived From Bovine Milk in the Dr. Kenneth Kaunda District, South Africa

**DOI:** 10.1155/ijm/7241123

**Published:** 2026-08-27

**Authors:** Lydia C. Sizana, Oriel Thekisoe, Kgaugelo E. Lekota, Tsepo Ramatla

**Affiliations:** ^1^ Unit for Environmental Sciences and Management, North-West University, Potchefstroom, South Africa, nwu.ac.za; ^2^ Centre for Applied Food Safety and Biotechnology, Department of Life Sciences, Central University of Technology, Bloemfontein, South Africa, cut.ac.za

**Keywords:** antimicrobial resistance, bovine milk, *E. coli*, virulence genes

## Abstract

The growing threat of antimicrobial resistance (AMR) among foodborne pathogens poses a significant public health risk in South Africa, largely driven by excessive antibiotic use in dairy farming practices. This study investigated the virulence and AMR profiles of *Escherichia coli* isolated from bovine quarter milk samples in the Dr. Kenneth Kaunda District, North West Province, South Africa. Sixty‐five *E. coli* isolates were confirmed using the automated identification Sensititre ARIS 2X ID/AST System and PCR targeting the *uidA* gene. Serogrouping revealed a predominance of non‐O157 strains, with O128 (50.8%) as the dominant serogroup, followed by O145 (9.2%), whereas 40% of isolates were nontypable, highlighting serotype diversity. The *fliC* gene encoding flagellin was the most prevalent virulence marker (62.5%), whereas *stx1* was detected in 9.2% of isolates, and *stx2* was absent. Phenotypic testing demonstrated high resistance to amoxicillin/clavulanic acid and cephalothin (58.9% each), tetracycline (37.8%), and trimethoprim–sulfamethoxazole (3.3%). However, no isolates exhibited phenotypic multidrug resistance (MDR), despite 98.5% harboring one or more resistance genes: *ermB* (92.3%), *vanB* (90.8%), *tetM* (56.9%), and *ampC* (10.8%). The high prevalence of *ermB* and *vanB* may indicate coselection of resistance determinants and suggests their potential association with mobile genetic elements that facilitate dissemination among bacterial populations. These findings indicate that *E. coli* from bovine milk in the region serve as silent reservoirs for virulence and resistance genes. These findings are vital for managing *E. coli* isolates in dairy farming and taking proactive measures to limit AMR in animals.

## 1. Introduction

Bovine mastitis is one of the costliest diseases in the dairy industry, resulting in substantial economic losses worldwide because of reduced milk yield, discarded milk due to high somatic cell counts, treatment costs, and the culling of infected cows [1, 2]. The severity of inflammation and the specific mastitis pathogen both influence the extent of milk yield loss. Infections from *Staphylococcus aureus* tend to be moderate but persistent, whereas infections caused by coliforms lead to more severe acute reductions in milk yield [3]. *Escherichia coli* is the most common coliform bacterium, causing the majority of coliform mastitis cases [4].

*E. coli* is a facultative anaerobic, gram‐negative, rod‐shaped, motile member of the Enterobacteriaceae found in the gastrointestinal tract of humans and warm‐blooded animals [5, 6]. Mastitis induced by *E. coli* may present as acute, clinical, or subclinical infections, sometimes progressing to severe systemic disease and irreversible damage to the mammary gland [7]. Severe *E. coli* clinical mastitis may cause irreversible damage to the mammary gland tissue, complete loss of lactation, and, at times, the death of a dairy cow [8]. *E. coli* has evolved into pathogenic variants that can cause various diseases, including bovine mastitis. Although the mammary gland is not a natural habitat for *E. coli*, this organism has acquired certain virulence factors that enable it to invade the mammary gland, survive, and multiply in milk [9].

Pathogenic *E. coli* strains are classified into several pathotypes, including enterotoxigenic *E. coli* (ETEC), enterohemorrhagic *E. coli* (EHEC), and enteropathogenic *E. coli* (EPEC), each defined by specific virulence traits [10]. Mammary pathogenic *E. coli* (MPEC) is increasingly recognized in mastitis cases [9–11]. Key virulence determinants include adhesins (*fimH*, *papG*), toxins (*stx1*, *stx2*, *hlyA*), and factors involved in attachment and invasion (*eae*, *kps*) [10, 12, 13].

Shiga toxin–producing *E. coli* (STEC) represents an important zoonotic group with variable pathogenicity [14]. Major non‐O157 serogroups include O26, O45, O103, O111, O121, and O145, which are linked to foodborne disease [15]. The pathogenicity of STEC is primarily driven by Shiga toxins and additional virulence factors such as intimin and hemolysins [16, 17]. These genes (*stx1*, *stx2*, and *eae*) are among the most critical determinants in mastitis‐associated *E. coli* [7].

Antimicrobial therapy is widely used for the management of mastitis in dairy systems [18]. However, the increasing prevalence of multidrug‐resistant (MDR) *E. coli* is a growing concern due to the misuse of antibiotics [19]. Resistance to critical antibiotics, including third‐generation cephalosporins and aminoglycosides, is mediated by *β*‐lactamases and aminoglycoside‐modifying enzymes, respectively [20]. *E. coli* harbors a variety of antibiotic resistance genes (ARGs), which include carbapenemase genes (*bla*_KPC_, *bla*_NDM_, and *bla*_OXA-48_), extended‐spectrum beta‐lactamase (ESBL) genes (*bla*_SHV_, *bla*_CTX-M_, and *bla*_TEM_), plasmid‐mediated quinolone resistance (PMQR) genes (*qnrA*, *qnrB*, *qnrS*, *qnrC*, *qnrD*, *qepA*, and *aac(6)-Ib-cr*), and tetracycline resistance genes (*tetA* and *tetB*). Furthermore, there are genes encoding for resistance to macrolides (*ermB*), sulfonamides (*sul1*, *sul2*, and *sul3*), trimethoprim (*dfrA*), aminoglycosides (*aac(3)*, *aph(3)*, and *aadA*), and chloramphenicol (*catA1*) [21].

Although studies in South Africa have reported the presence of *E. coli* and associated resistance in dairy systems [6], there is limited molecular‐level data linking serogroups, virulence factors, and resistance genes in specific regions. This gap is particularly evident in the Dr. Kenneth Kaunda District of the North West Province. Additionally, the lack of integrated genotypic and phenotypic analyses limits understanding of how even non‐MDR isolates may serve as reservoirs of resistance. Addressing this gap is essential for improving surveillance and guiding antimicrobial stewardship within a “One Health” framework. Therefore, this study focuses on characterizing the virulence and ARG profiles of *E. coli* isolated from bovine milk in the Dr. Kenneth Kaunda District, providing region‐specific baseline data to inform risk assessment and antimicrobial resistance (AMR) mitigation strategies.

## 2. Materials and Methods

### 2.1. Ethical Statement

Before the commencement of the study, approval was obtained from the Faculty of Natural and Agricultural Sciences Ethics Committee (FNASREC) at North‐West University, with the reference number NWU‐01306‐22‐A9.

### 2.2. Sample Size Determination and Sample Collection

A descriptive cross‐sectional study using convenience sampling was conducted. This was because of the unavailability of updated census data of dairy farms and dairy cattle in the Dr. Kenneth Kaunda District, which was necessary to determine a sample size representative of the population. A total of seven dairy farms with herd sizes ranging from 4 to 220 dairy cows were recruited for the study [22, 23]. These dairy farms were readily accessible as they were registered under the Brucella Milk Ring Test scheme at the state veterinary offices in the three municipalities within the district. All lactating cows were included in the study, regardless of age, breed, and number of lactations. The current study was conducted at the microbiology laboratories of the North‐West University in Potchefstroom, South Africa, between August 2023 and November 2024. A total of 517 dairy cows were accessible for sampling from the participating farms. However, only 441 could be sampled, as 76 were not in the lactation stage. Sample collection occurred between August 2023 and November 2024. The total number of lactating dairy cows to be sampled was determined based on the number of cattle per farm using a stratified sampling formula as shown in Table S1.

A total of 1730 quarter milk samples were collected from 441 lactating dairy cows (34 teats were nonproducing). Of these, 1720 were normal, and 10 showed clinical signs of mastitis. Samples were collected aseptically: Udders were washed, disinfected with 70% alcohol, and dried. Milk (20–40 mL) was collected in sterile bottles, stored in a cooler, and transported to the lab within 24 h. Samples were stored at 5°C ± 3°C to preserve their quality and integrity and tested within 48 h [23].

### 2.3. Isolation and Identification of *E. coli*

A milk sample (10 mL) was enriched in buffered peptone water (BPW Oxoid, Biolab, South Africa) (90 mL) and was incubated at 37°C for 24 h. Enriched samples were inoculated on MacConkey agar (MCA) and incubated at 37°C for 24–48 h. Pink colonies were considered presumptive *E. coli*. Single colonies were then purified on nutrient agar (Neogen, Scotland, United Kingdom). The *E. coli* ATCC 25922 and *S. aureus* ATCC 25923 reference strains were used as positive and negative controls, respectively. Pure colonies were transferred into buffered peptone water, and 1 mL of the pure colonies was transferred to a 30% glycerol solution (EMD Chemicals, United States) and stored at −20°C for future use.

### 2.4. Identification of *E. coli* Using the Sensititre System

Bacterial colonies were cultured on nutrient agar, incubated overnight at 37°C, and then emulsified in sterile saline to achieve a 0.5 McFarland standard turbidity. The suspension was dispensed into a gram‐negative Sensititre panel and incubated at 37°C for 18–24 h. Biochemical reactions were recorded and compared against the Sensititre database for species identification [24].

### 2.5. Genomic DNA (gDNA) Extraction

gDNA was extracted from overnight cultures of the 76 presumptive *E. coli* isolates incubated in Luria‐Bertani (LB) broth at 37°C. gDNA was extracted using the Qiagen DNeasy Blood & Tissue Kit (Qiagen, Germany) according to the manufacturer′s instructions. The quantity and quality of the gDNA were evaluated using a NanoDrop ND‐1000 spectrophotometer (NanoDrop Technologies, Wilmington, United States). The DNA samples were subsequently stored at −20°C until further analysis.

### 2.6. Genetic Identification of *E. coli*

A PCR assay was performed to confirm the identity of phenotypically identified *E. coli* isolates targeting the housekeeping *uidA* (*β*‐d‐glucuronidase) gene, as described by Ramatla et al. [25]. The primers used were UidA‐F AAA ACG GCA AGA AAA AGC AG and UidA‐R ACG CGT GGT TAC AGT CTT GCG, which produce a 147‐bp amplicon. The PCR mix contained 12.5 *μ*L of DreamTaq Green Master Mix, 8.5 *μ*L of RNase–nuclease‐free water, 1 *μ*L of each 10 *μ*M primer, and 2 *μ*L of the gDNA template, which were added to complete the mix. The PCR cycling process began with an initial denaturation step at 95°C for 10 min. This was followed by 35 cycles of 95°C for 45 s, annealing at 59°C for 30 s, and extension at 72°C for 90 s. A final extension was performed at 72°C for 10 min using the ProFlex PCR System (Applied Biosystems, United States). A DNA‐free template (nuclease‐free water) was used as a negative control.

To ensure standardization, molecular weight markers (Promega, Madison, Wisconsin, United States) of 1 kb and 100 bp were used to determine the sizes of the PCR amplicons. To verify the product size and yield estimation, 5 *μ*L of the PCR products was loaded onto a 1% agarose gel, electrophoresed for 40 min at 80 volts, stained with ethidium bromide, and then visualized under UV light.

### 2.7. Detection of Virulence Genes

PCR was used to detect STEC by targeting specific virulence genes. This included the *stx1* and *stx2* genes, *it* gene of ETEC, the enteroinvasive *E. coli* (EIEC), the *vir* gene related to EIEC, the hemolysin gene (*hlyA*), and the *aafII* gene linked to enteroaggregative *E. coli* (EAEC). The PCR assay and primer sequences used for the confirmation are summarized in Table 1. The reaction was carried out as described by Ramatla et al. [26] with a total volume of 25 *μ*L. The PCR conditions consisted of an initial denaturation at 96°C for 4 min, followed by 30 cycles of denaturation at 94°C for 30 s, annealing at 55°C–64°C for 30 s, and extension at 72°C for 1 min, using the ProFlex PCR thermocycler (Applied Biosystems, United States). Negative control was included by replacing the test DNA with 5 *μ*L of sterile nuclease‐free water.

**Table 1:** Primer sequences used to detect O‐serogroups and virulence genes of *Escherichia coli.*

Pathotype and O‐serogroup	Primer	Sequence of primers (5 ^′^–3 ^′^)	Amplification length (bp)	Annealing temp (°C)	Reference
*lt*	lt‐F	GCACACGGAGCTCCTCAGTCTCC	218	56	[26]
	Lt‐R	TTCATCCTTTCAATGGCTTT			
*aafII*	aafIIF	CACAGGCAACTGAAATAAGTCTGG	378	56	[27]
	aafIIR	ATTCCCATGATGTCAAGCACTTC			
*hlyA*	hlyA_F	GCATCATCAAGCGTACGTTCC	534	65	[28]
	hlyA_R	AATGAGCCAAGCTGGTTAAGCT			
*flic*	flicF	AGCTCAGGCAATGAAACTTTGAC	461	63	[29]
	flicR	GAAGCATACCCGGCAACAG			
*vir*	virF	AGCTCAGGCAATGAAACTTTGAC	822	61	[30]
	virR	TGGGCTTGATATTCCGATAAGTC			
*stx1*	stx‐1‐F	CAACACTGGATGATCTCAG	349	55	[31]
	stx‐1‐R	CCCCCTCAACTGCTAATA			
*stx2*	stx‐2‐F	ATCAGTCGTCACTCACTGGT	110	55	[31]
	stx‐2‐R	CTGCTGTCACAGTGACAAA			
O113	O113F	GGGTTAGATGGAGCGCTATTGAGA	771	58	[32]
	O113R	AGGTCACCCTCTGAATTATGGCAG			
*0157*	O157F	CGGACATCCATGTGATATGG	259	58	[32]
	O157R	TTGCCTATGTACAGCTAATCC			
*0103*	O103F	TTGGAGCGTTAACTGGACCT	321	58	[32]
	O103R	GCTCCCGAGCACGTATAAG			
*0145*	O145F	CCATCAACAGATTTAGGAGTG	609	58	[32]
	O145R	TTTCTACCGCGAATCTATC			
*0128*	O128F	GCTTTCTGCCGATATTTGGC	289	58	[33]
	O128R	CCGACGGACTGATGCCGGTGATT			

### 2.8. Antimicrobial Susceptibility Testing

Phenotypic antimicrobial susceptibility testing of isolates was performed by the disk diffusion method on 90‐mm agar plates [33]. It was conducted according to the protocols outlined by the Clinical and Laboratory Standards Institute (CLSI) guidelines (CLSI: M31‐A3, 2008; VET01S, 2020; M100, 2020). The antimicrobials included in the testing panels were gentamycin (10 *μ*g), amoxicillin/clavulanic acid (30 *μ*g), ticarcillin (75 *μ*g), cephalothin (30 *μ*g), trimethoprim/sulfa (25 *μ*g), and tetracycline (30 *μ*g) (Thermo Fisher Scientific, South Africa). A standardized inoculum (100 *μ*L) was applied to the surface of Mueller–Hinton agar (MHA). According to the CLSI, the zone of inhibition for antibiotics was measured in millimeters. For quality control in the antimicrobial susceptibility test, *E. coli* ATCC 25922 was used. Isolates exhibiting resistance to three or more antimicrobial classes were categorized as MDR [34, 35].

### 2.9. Detection of ARGs

A total of 13 ARGs were screened from 65 *E. coli* strains isolated from bovine milk. The AMR genes investigated included those that confer resistance to aminoglycosides (*strA* and *aadA*), tetracyclines (*tetK*, *tetM*, and *tetS*), quinolones (*aac(6*  ^′^ *)-Ib-cr*), *β*‐lactamases (*bla*_SHV_, *bla*_OXA_, *bla*_TEM_, and *ampC*), vancomycin (*vanA* and *vanB*), and macrolides (*ermB*). Reactions of 25 *μ*L were prepared, containing 12.5 *μ*L of DreamTaq PCR Master Mix (2X) (New England Biolabs, United States), 2.5 mM of each primer (Table 2), 2 *μ*L of DNA template, and double‐distilled water (ddH_2_O) to reach the final volume.

**Table 2:** Primer sequences used to detect selected antibiotic resistance genes in *Escherichia coli* serogroups detected from bovine milk.

Target gene	Primer	Sequence of primers (5 ^′^–3 ^′^)	Amplification length (bp)	Annealing temp (°C)	Reference
*aac(6* ^′^ *)-Ib-cr*	aac(6 ^′^)‐Ib‐cr‐F	ATATGCGGATCCAATGAGCAACGCAAAAACAAAGTTAG	544	66	[36]
	aac(6 ^′^)‐Ib‐cr‐R	ATAGCGAATTCTTAGGCATCACTGCGTGTTCGCT			
*tet(S)*	tet(S)	GAAAGCTTACTATACAGTAGC	169	50	[37]
	tet(S)	AGGAGTATCTACAATATTTAC			
*tet*(K)	tet(X)‐F	TCGATAGGAACAGCAGTA	169	61	[38]
	tet(X)‐R	CAGCAGATCCTACTCCTT			
*tet*(M)	tet(M)‐F	ACAGAAAGCTTATTATATAAC	171	60	[37]
	tet(M)‐R	TGGCGTGTCTATGATGTTCAC			
*bla*_TEM_	TEM‐F	TTC TTG AAG ACG AAA GGG C	1150	55	[39]
	TEM‐R	ACGCTCAGTGGAACGAAAAC			
*bla*_SHV_	SHV‐F	CACTCAAGGATGTATTGT G	885	55	[39]
	SHV‐R	TTAGCGTTGCCAGTGCTCG			
*bla*_OXA_	OXA‐F	ACACAATACATATCAACTTCGC	813	55	[30]
	OXA‐R	AGTGTGTTTAGAATGGTGATC			
*str*A	strA‐F	CTTGGTGATAACGGCAATTC	548	55	[37]
	strA‐R	CCAATCGCAGATAGAAGGC			
*aad*A	aadA‐F	ATCCTTCGGCGCGATTTTG	283	56	[37]
	aadA‐R	GCAGCGCAATGACATTCTTG			
*amp*C	AmpC‐F	GTGACCAGATACTGGCCACA	822	61	[37]
	AmpC‐R	TTACTGTAGCGCCTCGAGGA			
*VanA*	VanAst‐F	GAAATCAACCATGTTGATGTAGCA	572	59	[37]
	vanAst‐R	TTTGCCGTTTCCTGTATCCGT			
*vanB*	vanB‐F	CGCCATACTCTCCCCGGATAG	667	61	[40]
	vanB‐F	AAGCCCTCTGCATCCAAGCAC			
*erm*B	ERMB‐F	GCATTTAACGACGAAACTGGCT	573	61	[41]
	ERMB‐R	GACAATACTTGCTCATAAGTAATGGT			

The PCR protocol outlined by Ramatla et al. [26] consisted of an initial denaturation step at 95°C for 10 min, followed by 35 cycles of 95°C for 30 s, annealing at temperatures ranging between 50°C and 62°C (Table 2) for 30 s, elongation at 72°C for 30 s, and a final elongation at 72°C for 1 min. After amplification, the amplicons were analyzed by gel electrophoresis on a 1% (w/v) agarose gel stained with ethidium bromide, and the results were visualized under UV light using an ENDURO GDS Gel Documentation System (Labnet International Inc., United States).

### 2.10. Data Analysis

Statistical analysis was performed using Microsoft Excel 2016 (Microsoft Corporation, Redmond, Washington, United States) and Statistical Package for the Social Sciences Version 26 (IBM Corporation, Armonk, New York, United States). ChiPlot (https://www.chiplot.online/#) was used to generate heatmaps of the virulence and antibiotic resistance profiles.

## 3. Results

### 3.1. Genetic Identification of *E. coli*

Of the 76 presumptive *E. coli* isolates examined in this study, only 65 (85%) were confirmed by the Sensititre system and PCR using the *uidA* gene. The confirmed isolates produced the expected 147‐bp bands, which aligned with the positive control. No band was observed in the negative control, thereby confirming the validity of the test results (Figure S1).

### 3.2. Virulence Genes

**Figure 1:**
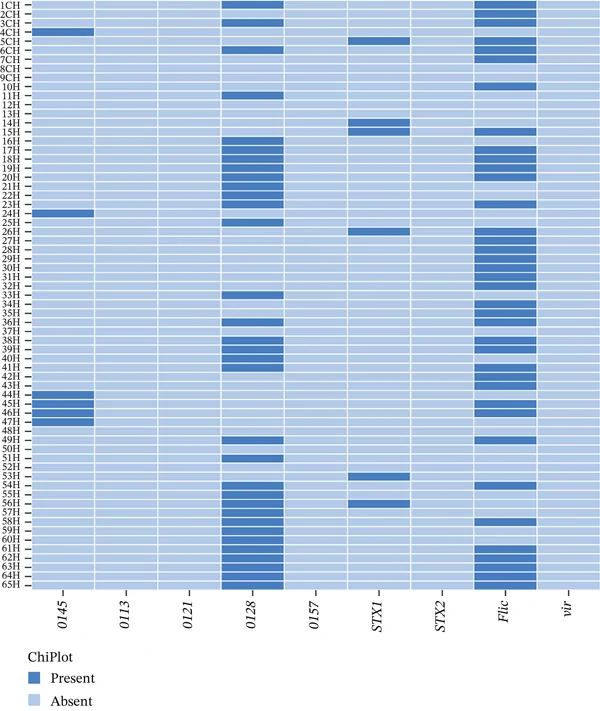
Distribution of *Escherichia coli* serogroups and virulence‐associated genes among the 65 isolates derived from bovine milk collected from dairy farms in the Dr. Kenneth Kaunda District. Blue indicates the presence of serogroups and virulence, whereas light blue represents the absence of serogroups and virulence (https://www.chiplot.online/).

The distribution of serogroups among the 65 isolates determined showed that *E. coli* O128 was the most prevalent (50.8%; *n* = 33), followed by *E. coli* O145 (9.2%; *n* = 6). Twenty‐six isolates (40.0%) remained nontyped (NT), and O113, O121, and O157 were not detected. The prevalence of Shiga toxins among the 65 isolates was low; *stx1* was present in 9.2% (*n* = 6) of isolates (83.3% [*n* = 5] NT, 16.7% O128), whereas *stx2* and *vir* were negative in all samples tested (100%). The *fliC* gene was present in 58.5% (*n* = 38) of isolates, distributed among 20 O128 isolates (52.6%), 2 O145 isolates (5.3%), and 16 NT isolates (42.1%). Seven NT isolates (10.7%) carried both *fliC* and *stx1* (Figure 1).

### 3.3. Phenotypic and Genotypic Antibiotic Resistance

**Figure 2:**
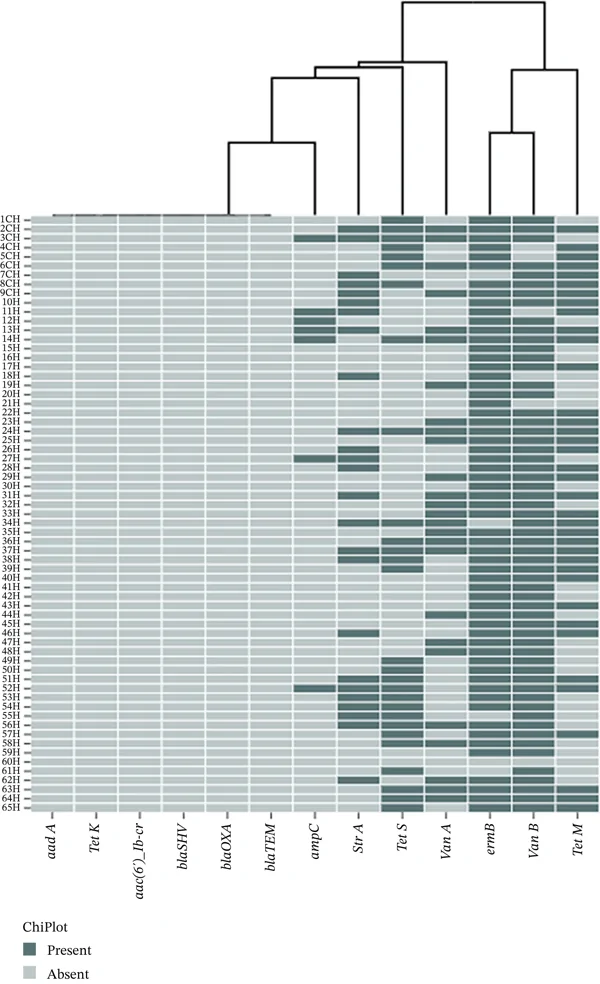
Distribution of antimicrobial resistance genes and hierarchical clustering of *Escherichia coli* isolates originating from bovine milk collected from dairy farms in the Dr. Kenneth Kaunda District. Dark green indicates the presence of an antibiotic resistance phenotype, whereas light green represents the absence of antibiotic resistance genes (https://www.chiplot.online/).

The phenotypic susceptibility testing indicated high‐level baseline resistance to amoxicillin/clavulanic acid and cephalothin (58.9% each), moderate‐level baseline resistance to tetracycline (37.8%), and low‐level baseline resistance to trimethoprim–sulfamethoxazole (3.3%) and ticarcillin (2.2%). Gentamicin showed 0% resistance. Genotypic profiling, however, indicated that 98.5% of the isolates possessed at least one resistance determinant. Resistance genes from 7 of 13 candidate genes were successfully amplified. The macrolide resistance gene *ermB* was expressed by the greatest number of isolates (92.3%), followed by the glycopeptide resistance marker *vanB* (90.8%). Tetracycline resistance genes *tetM* and *tetS* were also expressed by 56.9% and 43.1% of isolates, respectively. *vanA*, *strA*, and *ampC* were expressed by 40.0%, 38.5%, and 10.8% of isolates, respectively (Figure 2). Of note, although 70.8% of isolates possessed an MDR genotype with resistance to three or more classes of ARGs, none exhibited an MDR phenotype (carrying ≥ 3 classes).

## 4. Discussion

This study evaluated the virulence and AMR profiles of *E. coli* isolated from bovine milk in the Dr. Kenneth Kaunda District, North West Province, South Africa. All presumptive isolates were successfully confirmed as *E. coli* through the detection of the *uidA* gene. Although *uidA* is a reliable marker, its presence in *Shigella* species necessitates careful primer selection to ensure specificity [42]. Notably, the isolates were phenotypically non‐MDR yet harbored multiple resistance and virulence genes, highlighting their potential role as reservoirs of AMR within dairy production systems.

### 4.1. *E. coli* Serotypes and Virulence Genes

The absence of the EHEC O157 strain, often detected in food products, and the near‐monoclonality of O128 (50.8%) suggest either displacement of O157 STEC from cattle milk microbiota or a separate microepidemiology in this compartment. Regardless, this finding reflects the clonal community observed at the global scale with an epidemiological shift away from O157 STEC toward non‐O157 phylogenetic groups worldwide, described in surveillance and outbreak studies [26, 43, 44]. This global trend can, however, experience some geographic specificity at the level of raw milk (regional outbreak of O15, O99, and O88 serogroups in Korea, for example) [20]. In our study, O145 remained persistently detected, albeit at a low level (9.2%), allowing this medically important non‐O157 STEC serogroup to be represented in the shed population [45–47].

The overwhelming prevalence of O128, previously defined under the umbrella of the EPEC pathotype, suggests that this serogroup may be more commonly associated with intestinal colonization and enteric disease than with highly invasive STEC‐associated illness. However, this interpretation should be confirmed through further genomic characterization and pathogenicity studies [48]. This assumption is supported by the continued prevalence of mild pathotypes, as evidenced by O145. This highly virulent lineage remains present at baseline levels of low‐level contamination commonly found in raw milk [49]. Although concerning, the inability to type 40% of bovine‐derived milk isolates reflects the critical structural flaw of targeted‐serogroup PCR assays [50]. This finding highlights the genetic diversity of bovine‐associated *E. coli* populations and emphasizes the limitations of targeted serotyping approaches [51–53]. Broader genomic analyses, including whole‐genome sequencing (WGS), would be valuable for further characterizing these untyped isolates and determining their epidemiological significance [54].

In this study, only *stx1* was detected, with no evidence of the more virulent *stx2* gene. At a mechanistic level, given that lambdoid bacteriophages encode Shiga toxins, this separation suggests either a strong host restriction on phages carrying *stx2* or that there are no stable lysogens for *stx2* phages in the local O128 and O145 populations. The complete absence of *stx2* was found to be significantly associated with important clinical and public health implications. Whereas *stx1* has been associated almost exclusively with self‐resolving episodes of diarrhea [55], *stx2* has been shown to bind with greater affinity to receptors expressed on human kidney microvascular endothelial cells. Therefore, a lack of *stx2* suggests a reduced risk of developing more invasive sequelae, largely caused by *stx2* variants alone or in combination with *stx1*, such as hemolytic uremic syndrome (HUS) [56]. Furthermore, although only one O128 strain retained *stx1*, the majority of strains (58.5%) retained *fliC*, the gene encoding flagellin, as their sole virulence factor. This observation strongly suggests that flagellar assembly serves a more immediate role in localized intestinal colonization/adhesion events in the bovine udder rather than as part of a concerted acute cytotoxic response.

### 4.2. Phenotypic and Genotypic Antibiotic Resistance

Phenotypic analysis revealed high resistance to *β*‐lactams (58.9%) and moderate resistance to tetracycline (37.8%), with minimal resistance to trimethoprim/sulfamethoxazole (3.3%). These findings align with earlier studies reporting high resistance rates to *β*‐lactams and tetracyclines in *E. coli* strains sourced from bovine milk, though resistance rates varied [17, 56]. Drugea et al. [57] studied antibiotic resistance in *E. coli* from raw bovine milk over 5 years. They found 88.5% resistant to *β*‐lactams (penicillin and novobiocin) and 54.7% resistant to streptomycin, whereas resistance to tetracycline was only 22.3%. A study on *E. coli* strains from bovine milk samples showed that 51.1% were resistant to ampicillin, 42.5% to cefazolin, and 38.1% to erythromycin, with no resistance to tetracycline [58]. In this study, resistance patterns varied between farms but remained consistent within individual farms, suggesting that farm‐level practices, including antimicrobial use and management strategies, drive resistance selection [59]. This supports the need for farm‐specific AMR surveillance and targeted interventions rather than generalized regional strategies. Identifying risk factors for AMR at the individual farm level is essential to target specific problem areas. This highlights the need for farm‐specific surveillance and tailored antimicrobial stewardship, rather than relying solely on regional measures.

Importantly, the finding that resistance patterns differ between operations but are similar within each operation suggests that countrywide epidemiological models may not be adequate to understand local selection pressures. On‐farm decisions, such as the number of treatments, dosage, and indications for which intramammary antibiotic treatments for mastitis are used, likely serve as the primary filters driving resistome selection. Because of the segregation of MIC changes between operations, countrywide, top‐down interventions could waste valuable biosecurity efforts. Rather, the findings highlight the need for operation‐specific AMR monitoring systems and intervention plans. Targeting risk factors at the operational level may be the only way to eliminate these local selection pressures and preserve allies in veterinary and human medicine.

The detection of the macrolide resistance gene *ermB* (92.3%) and the glycopeptide resistance genes *vanB* (90.8%) and *vanA* (40.0%) highlights a significant burden of atypical/dormant/reserved resistance determinants in these isolates. The *ermB* prevalence is consistent with baseline observations in environmental samples (88% in soil and 92% in feces) [60]; however, there was no opportunity to directly associate genotype with phenotype as macrolides were not included on the antimicrobial panel used. The widespread prevalence of glycopeptide resistance genes *vanA* and *vanB*, however, seems paradoxical. Glycopeptides are ineffective against the outer membrane (lipopolysaccharide layer) of gram‐negative rods. They are either completely banned or highly restricted for use in food animals because of toxicity [61]. Macrolides and glycopeptides are not indicated or used to treat mastitis caused by coliforms, so there is unlikely to be any direct selective pressure associated with their use. Instead, the consistent presence of these resistance genes indicates pressure from the coselection of these otherwise clinically irrelevant/resistant silent gram‐positive markers. These resistance genes reside on the same mobile genetic element (highly conjugative broad‐host‐range plasmids, transposons, and integrons) as another highly selected marker (such as *β*‐lactamase [*ampC*] or tetracycline resistance [*tetM/S*]). As such, veterinary treatments on dairy farms using first‐line antimicrobials such as penicillins and tetracyclines to treat clinical cases of mastitis become the selective agent for these MDR genetic elements. Although this reservoir may be largely silent as the primary bovine *E. coli* host repertoires move from farm to farm, these highly mobile elements will encounter no species barriers to horizontal gene transfer events [62]. From there, they can easily be transmitted through food products and seed commensal human gut populations, serving as mutational breeding grounds for the transfer of these important resistance determinants to true pathogens [60].

Complete phenotypic susceptibility to gentamicin (100%) paired with the partial genotypic presence of *strA* (38.5%) suggests two local *E. coli* lineages are confronting this specific aminoglycoside selective pressure differently. Although gentamicin and streptomycin are both aminoglycosides, they are two chemically unique compounds that are enzymatically deactivated by distinct, highly specific mechanisms. Resistance to aminoglycosides is multifaceted; specific resistance determinants target different agents based on structural variation in the drug itself [63]. Because the aminoglycoside phosphotransferase [APH(3″)‐Ia] encoded by *strA* specifically phosphorylates streptomycin, there is likely no structural accommodation on this enzyme to recognize and alter the gentamicin core [64]. Additionally, phenotypic susceptibility to gentamicin should not be interpreted as the complete absence of a gentamicin‐specific resistome. Because our gene panel did not screen for the most prevalent genetic determinants of gram‐negative gentamicin resistance, including *aac(3)-II* and *aac(3)-IV* (aminoglycoside acetyltransferases) or *ant(2″)-Ia* (adenylyltransferase), we are unable to make genotypic–phenotypic associations with this antibiotic [65]. This aligns with the well‐known phenomenon that many cases of phenotypic resistance do not correspond to unidentified or silent portions of the genotypic resistome [66]. Therefore, although gentamicin can be used reliably in both clinical and veterinary settings in this population, WGS must be performed to confirm that silent or low‐abundance gentamicin‐modifying determinants are not present.

The epidemiological paradox between the relatively few genotypic occurrences of *ampC* (10.8%) and the phenotypic resistance to amoxicillin/clavulanic acid (58.9%) that we observed points to an intrinsically complex, multigenic foundation of *β*‐lactam resistance among these dairy populations. As *ampC* encodes a Class C cephalosporinase that confers resistance to early penicillins and cephalosporins, this low prevalence cannot explain the rampant clinical resistance observed in these populations. As such, this structural imbalance almost certainly denotes unidentified narrow‐ or broad‐ESBL genes or mutations that promote hyperproduction of inherent, unmapped *ampC* genes. Compared with countries that employ heavy selection/high‐intensity dairy farming practices, this genetic landscape is distinct. Recent bovine mastitis populations sampled in Iraq showed an equivalently high 75.8% amoxicillin/clavulanic acid resistance rate, supported by an overlapping 94.4% *ampC* prevalence [67].

On the other hand, these low baseline selections mirror those of low selection pressure environments—such as dairy farms in New Zealand relying on pasture‐based grazing—that had similarly low *ampC* frequencies of 3.3% in fecal samples and 8.3% in dairy wastewater [68]. Lastly, there is an interesting chemical paradox in the low level of ticarcillin resistance (2.2%) amid this high level of amoxicillin/clavulanic acid resistance. As clavulanic acid is meant to serve as a suicide inhibitor to protect amoxicillin from degradation by normal Class A *β*‐lactamases, a high resistance to amoxicillin/clavulanic acid with a related 100% susceptibility to ticarcillin suggests that the resistance mechanism is not due to enzymatic cleavage but rather an unmapped inhibitor‐resistant *β*‐lactamase (IRT) or a localized mutation in outer membrane porins that limits influx. Overall, these structural discrepancies illustrate that the use of a small, gene‐targeted panel leaves large gaps in our understanding and underscores the need to perform open‐ended genome sequencing to identify the spectrum of operable *β*‐lactamases.

High carriage of MDR genotypes (70.8%) with no phenotypic expression of multidrug resistance (0%) highlights a large potential for hidden or silent resistance genes present in this dairy community. The capture of MDR elements in conjunction with phenotypic susceptibility suggests that these genetic markers are not being expressed at the transcriptional level or are being repressed by host‐mediated forces [69]. Similar trends of multidrug resistance genes in bovine milk *E. coli* isolates have been reported [56, 57]. Regardless of phenotype, the prevalent resistances to both *β*‐lactams and tetracyclines observed should remain a public health and livestock concern, as their continued prevalence indicates strong selection pressures within regions that commonly use these antibiotic classes for treatment. Resistance selection on South African dairy farms is becoming an epidemic, driven in large part by local selective pressures arising from the misuse and overuse of antibiotics to treat and prevent mastitis in cattle herds. Moving forward, molecular identification is shifting away from elementary PCR assays and toward more complex comparative genomics and subtractive hybridization methods [70, 71]. Utilizing these suites enables the identification of mobile genetic elements that compose the resistome, the detection of promoter‐level gene suppression, and the discovery of new drug targets before latent genetic elements reach pan‐resistant phenotypes.

### 4.3. Prospects

This study employed targeted PCR screening to investigate selected virulence and AMR genes. Although this approach provided focused insights, it does not capture the full genetic landscape of the isolates. Future research will therefore incorporate WGS to enable comprehensive identification of resistance determinants, virulence factors, and mobile genetic elements. This genome‐wide approach will provide a deeper understanding of the molecular mechanisms underlying pathogenicity and resistance, ultimately supporting the development of more effective, evidence‐based antibiotic treatment strategies for bovine mastitis and contributing to efforts to mitigate AMR within dairy production systems.

## 5. Conclusion

This study provides region‐specific insights into the distribution of virulence and AMR genes among *E. coli* isolates recovered from bovine quarter milk samples in the Dr. Kenneth Kaunda District, North West Province, South Africa. Based on serogrouping and virulence gene profiles, the isolates appear to exhibit generally low pathogenic potential. However, the coexistence of multiple AMR genes highlights a significant risk for the persistence and dissemination of resistance within dairy production systems and along the food chain. These findings underscore the importance of integrated, evidence‐based interventions across animal, public, and environmental health sectors to combat AMR. Furthermore, they emphasize the need for continuous surveillance, strengthened biosecurity measures, and targeted antimicrobial stewardship practices to limit the emergence and spread of resistance in dairy environments.

## Funding

This study was supported by the National Research Foundation (10.13039/501100001321; CSUR23030681021).

## Ethics Statement

Ethical approval was obtained from the Animal Ethics Committee of the North‐West University (NWU‐01306‐22‐A9).

## Conflicts of Interest

The authors declare no conflicts of interest.

## Supporting information


**Supporting Information 1.** Additional supporting information can be found online in the Supporting Information section. Table S1: Sampling calculation. Figure S1: PCR amplification of *uidA* gene. Lane M: molecular weight marker (100 bp); lane −ve, negative control; lane +ve, positive control; Lanes 1–9 represent amplified isolates.

## Data Availability

All data generated or analyzed during this study are included in this article.
