# Supplementary material for: Genotypic Profiling of Virulence and Antibiotic Resistance Genes of Escherichia coli Isolates Derived From Bovine Milk in the Dr. Kenneth Kaunda District, South Africa
Source: Int J Microbiol. 2026 Aug 27;2026:7241123. doi: 10.1155/ijm/7241123 (PMC13522711; doi:10.1155/ijm/7241123)
Supplement: Supplementary file 1 [file IJM-2026-7241123-s001.docx]

**Genotypic profiling of virulence and antibiotic resistance genes of *Escherichia coli***

**isolates derived from bovine milk in the Dr Kenneth Kaunda District, South Africa**

**Lydia C. Sizana^1^, Oriel Thekisoe ^1^, Kgaugelo E. Lekota^1^, Tsepo Ramatla^2*^**

^1^ Unit for Environmental Sciences and Management, North-West University, Potchefstroom, 2531, South Africa.

^2^ Centre for Applied Food Safety and Biotechnology, Department of Life Sciences, Central University of Technology, 1 Park Road, Bloemfontein, 9300, South Africa.

*Corresponding author

Tsepo Ramatla, PhD

Tell: +27 51507 3911, Email: [tramatla@cut.ac.za](mailto:tramatla@cut.ac.za)

**Supplementary Table S1:** Sampling Calculation

N = Σ (N_i × (Z^2 × P_i × (1-P_i)) ÷ ((N_i-1) × E^2 + Z^2 × P_i × (1-P_i))

**1. Farm A sample size**

N_dairy cows = 15

P_dairy cows = 0.5 (if unknown, 0.5 is assumed for maximum variability)

E = 0.05

Z = 1.96

N = (15 x (1.96)2 x 0.5 x (1 - 0.5)) ÷ ((15 - 1) x (0.05)2 + (1.96)2 x 0.5 x (1 - 0.5))

= 14.5 ~ 15

**2. Farm B sample size**

N_dairy cows = 13

P_dairy cows = 0.5 (if unknown, 0.5 is assumed for maximum variability)

E = 0.05

Z = 1.96

N = (13 x (1.96)2 x 0.5 x (1 - 0.5)) ÷ ((13 - 1) x (0.05)2 + (1.96)2 x 0.5 x (1 - 0.5))

= 12.6 ~ 13

**3. Farm C sample size**

N_dairy cows = 220

P_dairy cows = 0.5 (if unknown, 0.5 is assumed for maximum variability)

E = 0.05

Z = 1.96

N = (220 x (1.96)2 x 0.5 x (1 - 0.5)) ÷ ((220 - 1) x (0.05)2 + (1.96)2 x 0.5 x (1 - 0.5))

= 140.1 ~ 140

**4. Farm D sample size**

N_dairy cows = 50

P_dairy cows = 0.5 (if unknown, 0.5 is assumed for maximum variability)

E = 0.05

Z = 1.96

N = (50 x (1.96)2 x 0.5 x (1 - 0.5)) ÷ ((50 - 1) x (0.05)2 + (1.96)2 x 0.5 x (1 - 0.5))

= 21.97 ~ 22

**5. Farm E sample size**

N_dairy cows = 4

P_dairy cows = 0.5 (if unknown, 0.5 is assumed for maximum variability)

E = 0.05

Z = 1.96

N = (4 x (1.96)2 x 0.5 x (1 - 0.5)) ÷ ((4 - 1) x (0.05)2 + (1.96)2 x 0.5 x (1 - 0.5))

= 3.97 ~ 4

**Supplementary Figure S1:** PCR amplification of *uidA* gene. Lane M: Molecular weight marker (100bp); Lane -ve, negative control; Lane +ve, positive control; Lane 1-9 represents amplified isolates.

.
